# Iterative prototyping based on lessons learned from the falloposcope *in vivo* pilot study experience

**DOI:** 10.1117/1.JBO.28.12.121206

**Published:** 2023-08-12

**Authors:** Andrew D. Rocha, William K. Drake, Photini F. Rice, Dilara J. Long, Hasina Shir, Ryan H. M. Walton, Mary N. Reed, Dominique Galvez, Taliah Gorman, John M. Heusinkveld, Jennifer K. Barton

**Affiliations:** a The University of Arizona, Wyant College of Optical Science, Tucson, Arizona, United States; b The University of Arizona, Biomedical Engineering Department, Tucson, Arizona, United States; c The University of Arizona, Clinical and Translational Services, Tucson, Arizona, United States; d The University of Arizona, Department of Obstetrics and Gynecology, Tucson, Arizona, United States

**Keywords:** microendoscope iterative design and prototyping, multispectral fluorescence imaging, endoscopic optical coherence tomography, *in vivo* imaging, ovarian cancer, fallopian tube

## Abstract

**Significance:**

High grade serous ovarian cancer is the most deadly gynecological cancer, and it is now believed that most cases originate in the fallopian tubes (FTs). Early detection of ovarian cancer could double the 5-year survival rate compared with late-stage diagnosis. Autofluorescence imaging can detect serous-origin precancerous and cancerous lesions in *ex vivo* FT and ovaries with good sensitivity and specificity. Multispectral fluorescence imaging (MFI) can differentiate healthy, benign, and malignant ovarian and FT tissues. Optical coherence tomography (OCT) reveals subsurface microstructural information and can distinguish normal and cancerous structure in ovaries and FTs.

**Aim:**

We developed an FT endoscope, the falloposcope, as a method for detecting ovarian cancer with MFI and OCT. The falloposcope clinical prototype was tested in a pilot study with 12 volunteers to date to evaluate the safety and feasibility of FT imaging prior to standard of care salpingectomy in normal-risk volunteers. In this manuscript, we describe the multiple modifications made to the falloposcope to enhance robustness, usability, and image quality based on lessons learned in the clinical setting.

**Approach:**

The ∼0.8  mm diameter falloposcope was introduced via a minimally invasive approach through a commercially available hysteroscope and introducing a catheter. A navigation video, MFI, and OCT of human FTs were obtained. Feedback from stakeholders on image quality and procedural difficulty was obtained.

**Results:**

The falloposcope successfully obtained images *in vivo*. Considerable feedback was obtained, motivating iterative improvements, including accommodating the operating room environment, modifying the hysteroscope accessories, decreasing endoscope fragility and fiber breaks, optimizing software, improving fiber bundle images, decreasing gradient-index lens stray light, optimizing the proximal imaging system, and improving the illumination.

**Conclusions:**

The initial clinical prototype falloposcope was able to image the FTs, and iterative prototyping has increased its robustness, functionality, and ease of use for future trials.

## 1 Introduction

Ovarian cancer is the most deadly gynecological malignancy and is the fifth leading cause of cancer-related mortality in the United States in females, with incidence and mortality rates expected to be 19,880 and 12,810, respectively, in 2022. The early detection of ovarian cancer could double the 5-year survival rate compared with late-stage diagnosis (93% to 98% versus 31% to 45%, respectively).^1^^,^^2^ Recent evidence suggests that the molecular pathogenesis of high-grade serous ovarian cancer begins in the fallopian tubes (FT), prior to the onset of symptoms.^3^^–^^5^ Thus, there is an unmet clinical need for the earlier detection of pathological changes in the FTs. There is currently no generally acceptable screening method for ovarian cancer. Surveillance by annual concurrent transvaginal ultrasound and serum CA125 measurements are alternatives offered to women at very high risk for ovarian cancer who decline prophylactic salpingo-oophorectomy. Studies have revealed that this combination may be helpful but is insufficient, as only 50% to 60% of cases are diagnosed at early stages in these women and this method of screening is not recommended for women at average risk.^6^^–^^10^

Optical imaging has high resolution and sensitivity, which may offer the ability to detect ovarian cancer earlier than current screening methods. We and others have investigated optical methods for the early detection of ovarian cancer. Combined second harmonic generation microscopy with mass spectrometry has been shown to visualize differences in the collagen architecture between normal tissue and ovarian cancer precursors through proteomic analysis.^11^ High-optical-resolution photoacoustic microscopy reveals that malignant ovarian tissue has larger and tortuous blood vessels as well as smaller vessels of varying sizes, whereas benign and normal ovarian tissues have smaller vessels of uniform size.^12^ Fluorescence spectroscopy/imaging provides information on tissue composition, metabolism, and/or targeted contrast agents. Autofluorescence imaging can detect serous-origin precancerous and cancerous lesions in *ex vivo* FT and ovaries with good sensitivity and specificity,^13^ and multispectral fluorescence imaging (MFI) can differentiate healthy, benign, and malignant ovarian and FT tissues.^14^ Optical coherence tomography (OCT) reveals subsurface microstructural information and can distinguish normal and cancerous structure in ovaries and FT.^15^^–^^18^ Because fluorescence techniques provide functional information whereas OCT provides microstructural information, they are potentially synergistic modalities. They can both be easily implemented with small diameter fiber and micro-optics, making them well suited for FT imaging.

High resolution optical methods have a limited depth of imaging; therefore, a significant challenge is accessing the FTs in a minimally invasive manner. We developed a handheld sub-millimeter microendoscopy system, the falloposcope, as a minimally invasive method that can be implemented in an outpatient setting.^19^ The device hardware includes MFI at four visible wavelengths and OCT imaging for FT surveillance and differentiation between healthy, benign, and malignant tissue morphology. The falloposcope is a disposable device made with low-cost components designed to be universally used with standard hysteroscopes and introducing catheters for proximal FT catheterization. The current prototype is approved for sterilization with hydrogen peroxide vapor and all materials in possible contact with human tissue are fabricated with biocompatible materials. The falloposcope’s three terminated fibers are connected to a compact medical instrument cart permitted for use in the operating room (OR). This medical cart houses the MFI and OCT sources, MFI optics and camera, OCT detector, and the hardware for data acquisition, graphical user interface (GUI), and displays. These features not only enable FT surveillance but also could minimize the economic burden and improve global accessibility to ovarian cancer early detection.^20^

A clinical pilot study is currently underway to evaluate the device safety and to demonstrate the real-time OCT and MFI capabilities within a 15-min timeslot at Banner University Medical Center-Tucson in Tucson, Arizona, United States. The device is used for FT imaging prior to gynecological surgery in eligible and consented volunteers. Inclusion criteria include the following: subject is medically cleared for surgery, subject is scheduled to undergo a surgical procedure including a cervical dilation and salpingectomy, subject is being administered prophylactic antibiotics as part of surgery, subject must be at least 18 years of age, and subject must be able to provide informed consent. Exclusion criteria include the following: known high risk for ovarian cancer; acute pelvic inflammatory disease; active or recent lower pelvic infection; pregnancy; delivery or termination of a pregnancy in the past 6 weeks; known tubal obstruction including tubal ligation, invasive carcinoma of the cervix, or endometrium; or intolerance of anesthesia. This study has been designated non-significant risk by the Food and Drug Administration (FDA). Our study is approved for use on 20 patients by July 15, 2023. We have completed 12 pilot study procedures to date. Each pilot study has revealed successes, challenges, and feedback from operators and stakeholders, which have guided iterative improvements to the prototype.

The purpose of this manuscript is to present the improvements made to the falloposcope system through an iterative prototyping process, resulting in a more robust and user-friendly instrument with improved image quality. Iterative prototyping is a method that involves using stakeholder feedback to make improvements and gradually evolve a design and usability with each new prototype. Beginning with an initial prototype to demonstrate basic functionality, iterative prototyping brings the product concept to reality while synthesizing the components necessary to meet the needs of the application. It is important to engage multiple stakeholders, in our case experienced gynecologic surgeons, surgical support staff, medical device industry personnel, and ovarian cancer advocacy groups, to provide informed feedback on the device. This study incorporated qualitative and conceptual feedback while adhering to approved protocols, with utmost concern for patient safety. In light of complex anatomical differences in patients and variable surgical workflows and environments, we view iterative prototyping as the best method for improvement of the device design to better suit the diverse patient population with its naturally occurring variations in uterine and FT size, shape, and anatomy. It is our hope that, by sharing the operator feedback and design challenges that we have experienced throughout our iterative bench-to-bedside approach, we can provide valuable insight to engineers and scientists who design and build microendoscopes for clinical applications.

## 2 Initial Design and Pilot Study Protocol

The first benchtop prototype was a flexible, steerable microendoscope ∼0.9  mm in diameter.^21^ The next evolution of the clinical prototype involved moving the falloposcope systems on a portable platform that was qualified to be in a hospital OR. This system has been described previously^19^ and is summarized here. The falloposcope system can be subdivided into the OCT, MFI, workstation PC, and power systems. The OCT system has a swept source laser with a 20 kHz scan rate centered at 1300 nm that is coupled to an angled physical contact (APC) terminated single mode fiber (SMF). The interferometric fiber system splits the signal into the reference arm and the sample arm via a 2×2 fiber coupler. The reference arm includes a delay line for phase matching and a fiber optic retroreflector. The section of the sample arm in the cart ends with a ferrule connector (FC)/APC connector and is completed when the falloposcope’s OCT probe is connected. The return signal follows a path through a circulator that guides the signal into the other balanced photodetector port. The workstation PC takes in the balanced photodetector’s signal via a specialized data acquisition (DAQ) board.

The OCT’s system’s axial resolution is 10.7  μm, and its lateral resolution is ≤17  μm. The one-way transmission loss of an example endoscope is 1.6 dB; only a small portion of the lost power is back reflected into the system. With the endoscope connected but no reference mirror or sample, the total back reflected power at the detector is 0.320  μW, nearly all of which originates from the endoscope. Adding a mirror at the sample location provides a total back reflected power at the detector of 4.96 mW, giving a signal to noise ratio on the order of 15000. The back reflections are sufficiently low power to not cause detector saturation issues. Back reflections in the endoscope were minimized by ensuring that fiber splices were low loss and by setting the angle of the fold prism (angle polished fiber) to 48% so that back reflections from the prism exit surface and protective envelope were less likely to re-enter the endoscope’s SMF. The endoscope is also designed to work in an aqueous environment, which reduces back reflections from the outside surface of the protective envelope. The prism exit face could be antireflection coated in the future to further reduce back reflections. The strongest back reflection normally arises from the inside surface of the protective envelope. As this envelope is made from polyethylene terephthalate (PET) shrink tubing, it is impractical to the antireflection coat.

The pilot study protocol was reviewed and approved by the University of Arizona Institutional Review Board and follows HIPAA Privacy Program Guidance. Although FDA guidance states that falloposcopes are generally considered a significant risk, this falloposcope pilot study received a Nonsignificant Risk designation by the FDA. The laser output power on the tissue meets American National Standard for Safe Use of Lasers standard Z136.1 for exposure of skin. Laboratory Sciences of Arizona clinical infection prevention standards for sterilization of the single-use device before use with patients/tissue was followed by verification that there was no detectable *C. difficile* or *E. cloacae* bacteria after sterilization. The medical cart was verified to meet clinical electrical safety requirement IEC 60601-1 through the Banner Hospital Biomedical Engineering Department. All components of the endoscope that came into contact with the patient were deemed biocompatible per the manufacturer. This includes compliance with ISO 10993 for the adhesives and ISO 10993 and USP class VI compliance for the polymer materials.

The MFI system’s illumination consists of 405, 488, 520, and 642 nm fiber pigtailed laser diode sources with independent laser temperatures and current controllers. The laser diodes are pigtailed with SMFs with PC FCs. The FC/PC connectors of the sources are plugged into a beam combiner (Oz Optics, WDM-14P-11111-405/488/520/642-3/125,3.5/125,4/125,9/125-SSSSM-40-3S3S3S3S3A-1-1) that outputs the overlapped beams into one FC/PC terminated SMF. The SMF is secured to a plate that serves as the port for the endoscope’s illumination fiber to connect to. The falloposcope’s image fiber bundle is terminated with an SMA905 type connector. This connects to an image input port adjacent to the MFI illumination output. The input port is positioned behind an optical objective, which collimates the light to pass through a filter wheel and then focus down onto a scientific camera (Teledyne Photometrics, Retiga R6). The camera has a USB output that is taken by the workstation PC. The workstation handles the program that outputs the real-time display of the MFI and OCT systems as well as taking on the task of screen recording the procedures. The cart’s power is routed through an isolation trans-former (Tripp Lite, PN: IS1000HG, Chicago, Illinois, United States) to adhere to electrical safety standards of the hospital.

**Fig. 1:**
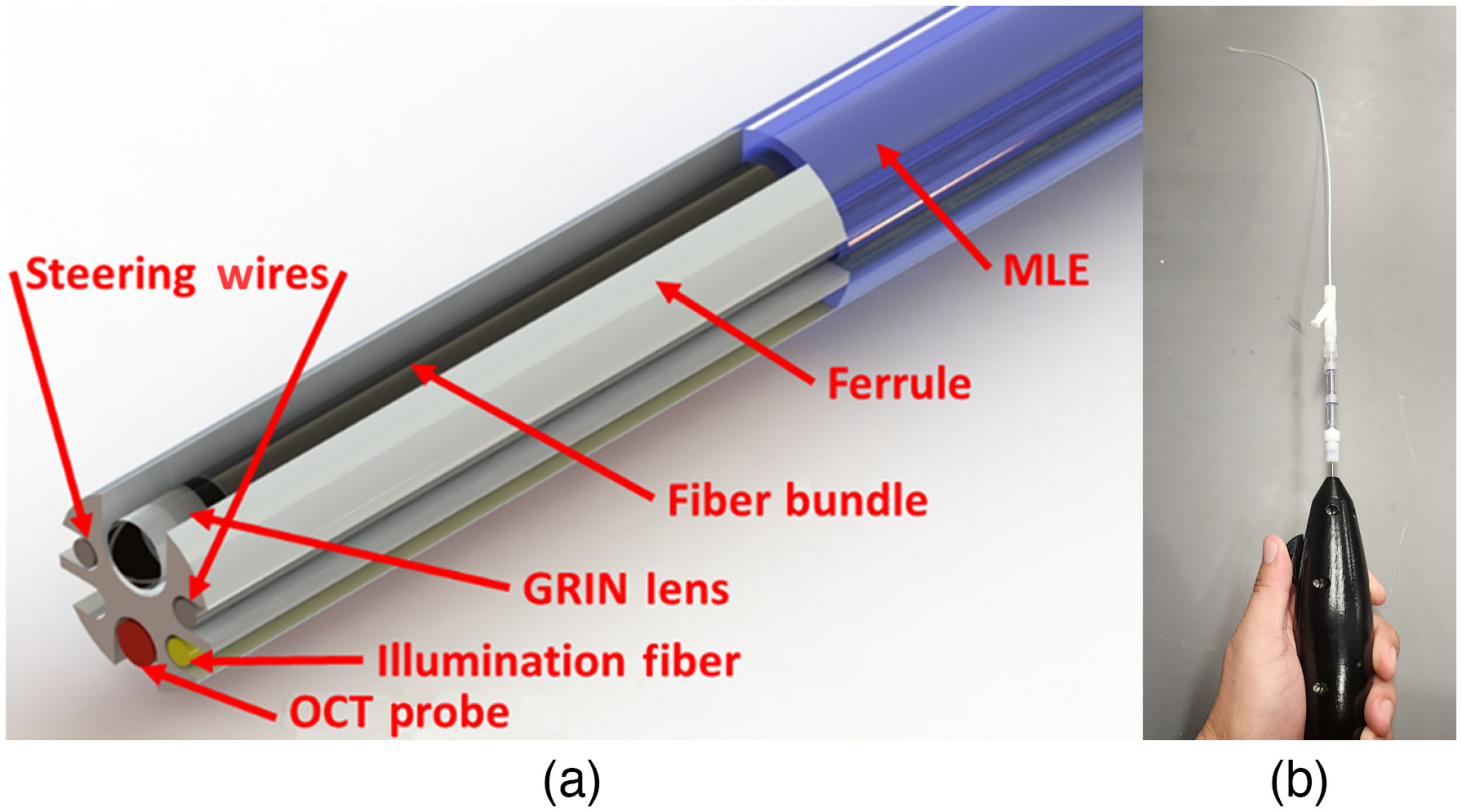
Clinical prototype falloposcope. (a) Before enveloping the working length of the falloposcope in heat shrink, the distal tip ferrule holds the fiber bundle, OCT fiber probe, illumination fiber, and pull wires that are threaded through the MLE. (b) The falloposcope is designed to be small enough to fit through a 5.5 Fr introducing catheter.

The clinical prototype falloposcope met all necessary specifications and performed well in laboratory testing, including tissue model imaging (SynDaver, SynTissue Uterus). The diameter of the insertable portion was decreased to ∼0.8  mm while substituting the single tube housing with a polyetheretherketone (PEEK) multilumen extrusion (MLE) with an outer PET heat shrink. The MLE holds a pull wire for steering, a 100  μm core diameter multimode fiber (MMF) for MFI illumination, a 3000-element fiber bundle and gradient-index (GRIN) lens for reflectance and fluorescence imaging, and an OCT probe. The clinical prototype is shown in Fig. 1.^19^ The clinical procedure is as follows: prior to salpingectomy, a hysteroscope with a 7F working channel (Olympus A4773 Sheath, A4674A telescope) and 5.5F selective salpingography (SSG) introducing catheter (Cook Medical, J-SSG-554086) are inserted through a dilated cervix, and the SSG catheter is hysteroscopically guided to the FT ostium. Once docked, the falloposcope’s working length is threaded through the SSG catheter and guided through the uterotubal junction. The falloposcope is then advanced to the proximal, middle, and distal portions of the FT for acquisition of four reflectance images, three fluorescence images, and a pullback OCT scan. If time permits, the procedure is repeated on the contralateral FT.

Our first procedures revealed several differences in the OR environment versus the laboratory. These included the bright illumination in the room, which exacerbated stray light issues, and the short time available, which necessitated that images be acquired as rapidly as possible without the manual adjustment needed to focus or make light source/camera adjustments. Additionally, the need to improve the image quality, simplify the system, and increase robustness was observed during these operations. For our falloposcope system to survive the rigors and urgency of real-time surgical environments, it had to be comparable in mechanical strength and simplicity of use to medical instruments with which the OR staff were already familiar. Thus, new developments and changes^22^ outlined below were made to improve the fabrication and functionality of the falloposcope and the instrument cart to increase ergonomic use by operators while increasing the image quality and mitigating the risk of malfunction during the procedure.

## 3 Pilot Study Lessons Learned

### 3.1 Operating Room Environment

Falloposcopes are submitted to the sterilization department the day before the surgery. On the day of the surgery, the falloposcope is delivered to the OR with all other medical instruments necessary for the gynecological procedure. While the surgical technicians prepare for about 30 min, the research team sets up the falloposcope medical cart. When the patient is transported into the room by hospital bed, they are transferred onto the operating table and then anesthetized. Our team is asked to vacate the OR during that time to give the patient privacy. Once the patient is fully anesthetized, we are allowed to continue setting up the medical cart. There are a few minutes until the surgeon begins the time out, during which the OR team reviews the patient’s identity, the procedure, and the surgical site before the start of the procedure. At the surgeon’s discretion, after cervical dilation and prior to salpingectomy, the pilot study is performed. After the pilot study procedure’s 15-min time is up, the surgeon continues with the procedure, and the research team waits to get explanted tissue and retrieve the pilot study instruments and tools that were used.

**Fig. 2:**
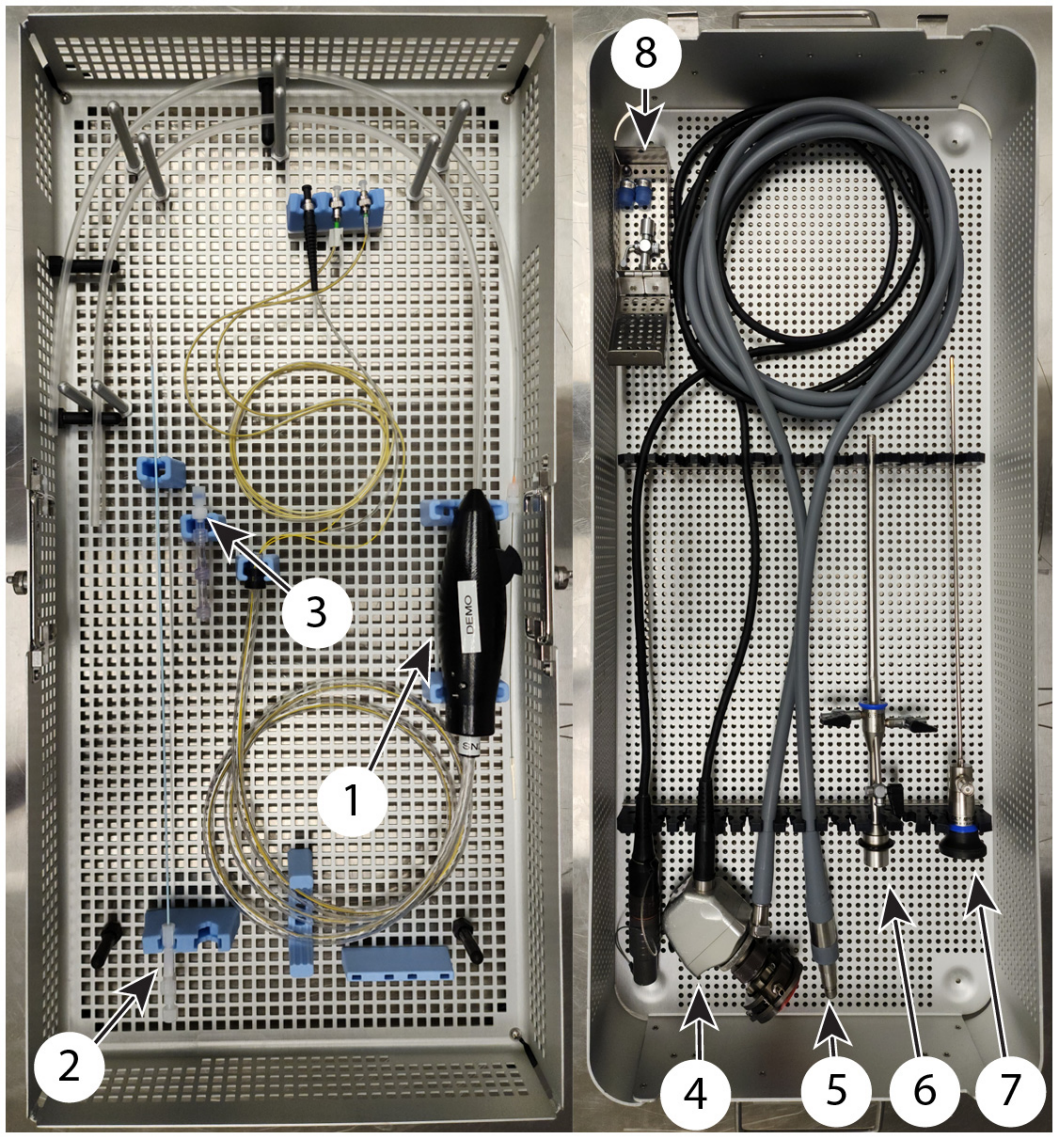
Left: sterilization case with disposable instrumentation, including the (1) falloposcope, (2) introducing catheter, and (3) introducing catheter components. Right: sterilization case with reusable instrumentation, including (4) hysteroscope camera, (5) light guide cable, (6) hysteroscope sheath, (7) hysteroscope telescope, and (8) additional necessary components.

We quickly learned that the varying sizes and configurations of the ORs, as well as surgical staff preference, meant that we needed to be flexible to accommodate any placement within the OR that was asked of the research team, requiring such minor but critical items as a long extension cord. Communications with staff, including clear directions from the surgeon, are also critical. For example, in first pilot study, although the research team was out of the OR, both the primary and secondary falloposcope cases were opened and placed on tables with other medical tools to be used in surgery. We were able to share a warning about the delicacy of the falloposcopes with one surgical technician, but as the procedure was about to start, more surgical technicians came in and the one who performed the preparations left. Communication at that point was difficult because all unnecessary chatter must end once time out commences. We anxiously watched our microendoscopes be handled roughly, be placed in precarious positions, and have other medical tools piled on top of them after the procedure. For the next procedure, we requested one table to be solely dedicated to the research team’s equipment. We asked that all of the components be carefully removed from the case and placed onto the table, and the case itself handed back to the research team to avoid inadvertent damage to the microendoscope. Figure 2 shows the arrangement of the falloposcope and accessory items (introducing catheter and hysteroscope) in the sterilization cases.

A second challenge was providing a clear view of falloposcope navigation using the forward facing MFI system with only the 488 laser source on. At the medical staff’s request, we did not connect our falloposcope instrument cart to the OR’s room monitors. Instead, a second opposite facing monitor was added to the cart to allow the surgeon to visualize MFI navigation and OCT scans. After the first subject was completed, the surgeon stated that the imaging system frames per second (fps) was not fast enough (about 7 fps) for them to comfortably navigate the microendoscope. The original software had a frame rate inversely proportional to the size of the display window, so either the image could be large enough or the frame rate fast enough to be comfortable to the physician, but not both. To increase the fps, we reduced the temperatures in the cart by 10°C to increase performance of the graphical processing unit and central processing unit while selecting a reduced window size to maintain a stable 20 fps live feed. To increase the video size in a large, comfortably viewed window, we used alternative software to magnify and stream the camera output to a second monitor. Display settings were optimized in the software and the monitor to enhance contrast. During the debrief after implementing this solution, the surgeon said that they were comfortable with the frame rate and were able to successfully navigate the microendoscope.

**Fig. 3:**
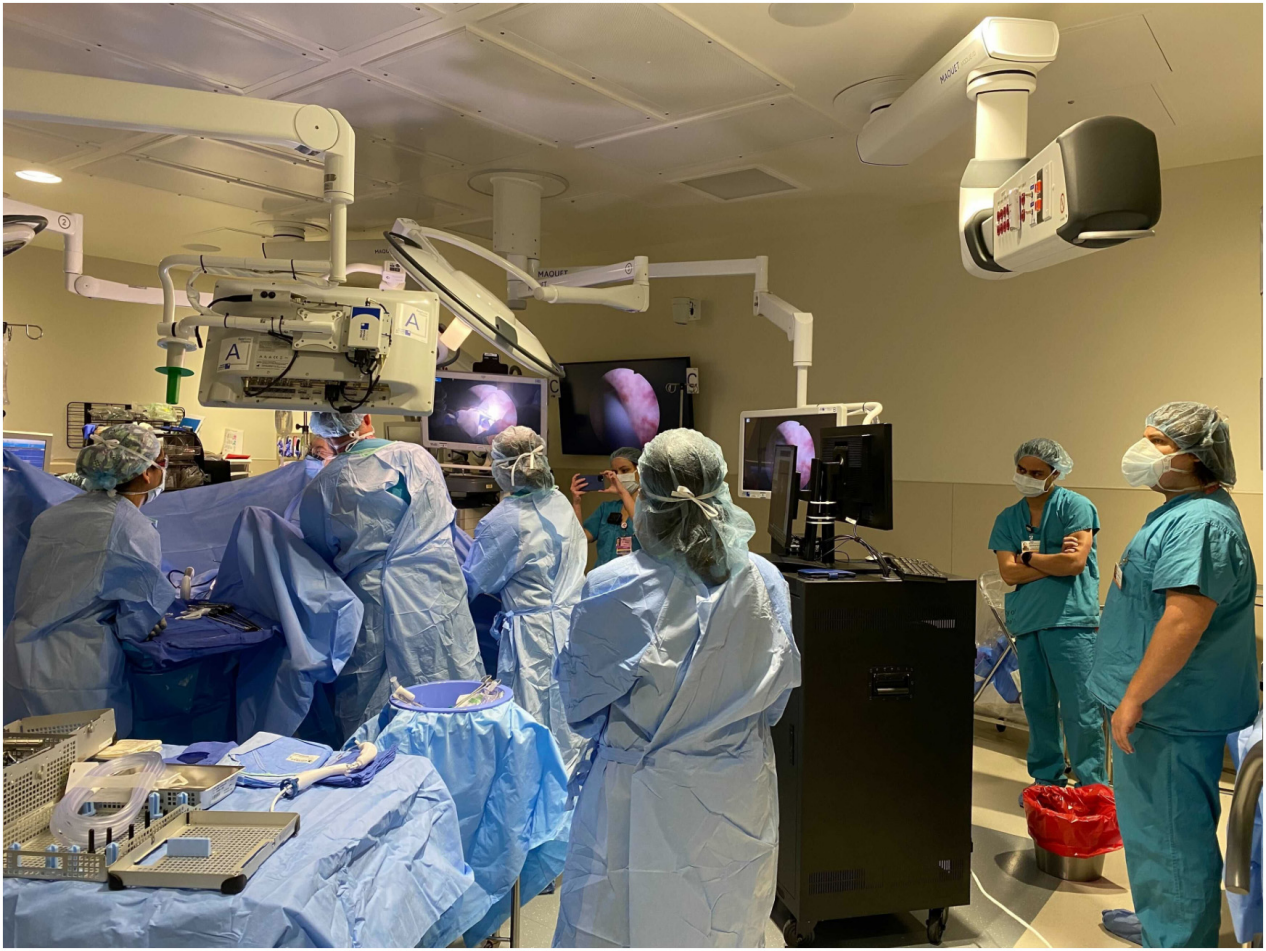
Attending physician performing a hysterectomy; the research team observes after our first successful 15-min pilot study procedure was completed. Sources and detectors are located in the black medical rack.

After a few patients, we realized that it was beneficial to record footage of the hysteroscope camera feed, which is not mandatory and even when obtained could be difficult to access after the procedure. We added our own hysteroscope camera module, which had two video outputs: a primary output connected to an external capture card to record the procedures and a secondary output plugged into a proprietary digital to analog converter to allow the display to be fed into the OR monitors. The configuration of the hysteroscopic view on the OR monitors and the falloposcopic view on the large instrument cart monitor were acceptable to the surgeon. Figure 3 shows the arrangement of the instrument cart in the OR.

### 3.2 Hysteroscope Accessories

**Fig. 4:**
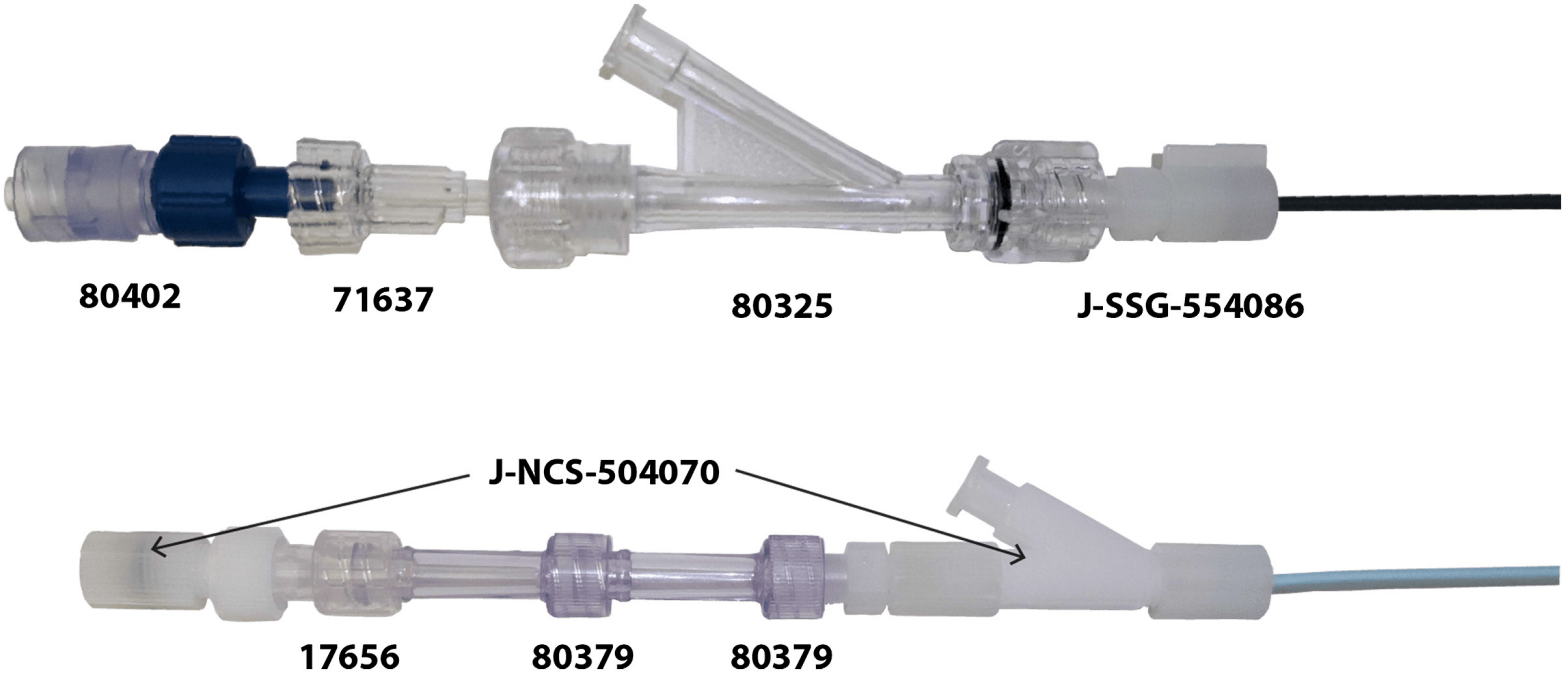
Introducing catheter components. Top: original configuration of a Tuohy Borst (Qosina 80402); Male Luer Lock Tubing Connector (Qosina 71637); and hemostasis valve y-connector (Qosina 80325), which connects to the 5.5 fr SSG catheter (Cook J-SSG-554086). Bottom: current configuration of a Tuohy Borst Adapter from the modified Novy cornual cannulation set, a female Luer lock to male Luer lock connector (Qosina 17656), two female Luer locks to male Luer lock connectors (Qosina 80379) connect to the 5.5 fr modified Novy cornual cannulation catheter (Cook J-NCS-504070) with included y-connector, inner catheter, and 0.35” guide wire.

Our first few pilot studies had SSG introducing catheters combined with commercial off-the-shelf single-use medical device components from Qosina (Ronkonkoma, Long Island, New York). We assembled these components consisting of a hemostasis valve y connector (Qosina 80325), Luer lock tubing connector (Qosina 71637), and Tuohy Borst valve adapter (Qosina, 80402), as shown in Fig. 4 (top), to enable saline flushing through the introducing catheter around the falloposcope inside. This assembly was determined to be suboptimal. After some falloposcope damage was noted, it was determined that interior stepped edges of the Tuohy Borst valve adapter and hemostasis valve y connector were catch points for the endoscope’s distal face. The hemostasis valve y connector was essential for the saline flush and prevention of backflow, but the Tuohy Borst valve adapter was not because its purpose was to lock the falloposcope into position, a functionality that the surgeon determined was not necessary. Moreover, the size and weight of the Qosina components that comprised the original introducing catheter assembly increased the difficulty of concurrently manipulating the hysteroscope, introducing catheter assembly, and falloposcope during the pilot study procedure. To complete the pilot study procedure, the surgeon had to enlist the aid of at least one other sterile person to manage use of all three independent instruments. The SSG catheter’s tip was opaque and difficulties in docking the tip to the ostium and having position awareness of the falloposcope’s tip were frustrating to the surgeon and surgeon’s aid. Our new configuration incorporates a modified Novy cornual cannulation set (Cook Medical J-NCS-504070). The Novy set already includes a y connector and has a clear tip, giving the surgeon better visual awareness of the tool’s position. A short female Luer lock to male Luer lock connector (Qosina 17656) and two longer female Luer lock to male Luer lock connectors (Qosina 80379) (Fig. 4, bottom) were added to lengthen the catheter assembly, enabling the Tuohy Borst to tighten abound the microendoscope’s semirigid tubing. An added advantage was the Novy set’s 0.035” diameter guide wire, which could be used to check patency in the FT prior to falloposcope introduction.

### 3.3 Endoscope Robustness/Fiber Breaks

In some procedures, we began with a fully functioning microendoscope, and by the time we returned to the lab to run *ex vivo* measurements, the microendoscope had lost functionality of one of the three major systems: OCT, illumination, or imaging. The two most common failures included breaks at the fiber splice junctions between the handle and instrument cart or at the rigid-to-flexible transitions of support material. In case of a structural failure of the primary falloposcope during sterilization, pre-operation setup, or the pilot study procedure, a back-up falloposcope was also sterilized and put on standby. However, the short time allotted for the pilot procedure (15 min) meant that switching out during the procedure was impractical. Stakeholders requested an increase in the robustness of the design. Because sterilization and human subjects approval were dependent on the exterior materials and sealing methods, major modifications in the falloposcope fabrication technique were limited as new, “from scratch” prototypes would have to be retested and reapproved.

In designing a more robust system, we can utilize commonly used fiber optic cable design practices based on telecommunication industry trends. Most field technicians that use fusion splicers in the field must use care and relieve potential future strain at the weak point by adding a splice protector with a junction box that holds the fiber’s strength member and fiber position in place to minimize tensile forces on the glass fiber. Our initial design tried to implement the fiber optic cable without strength members or a splice protector due to limited space and to minimize cost.

**Fig. 5:**
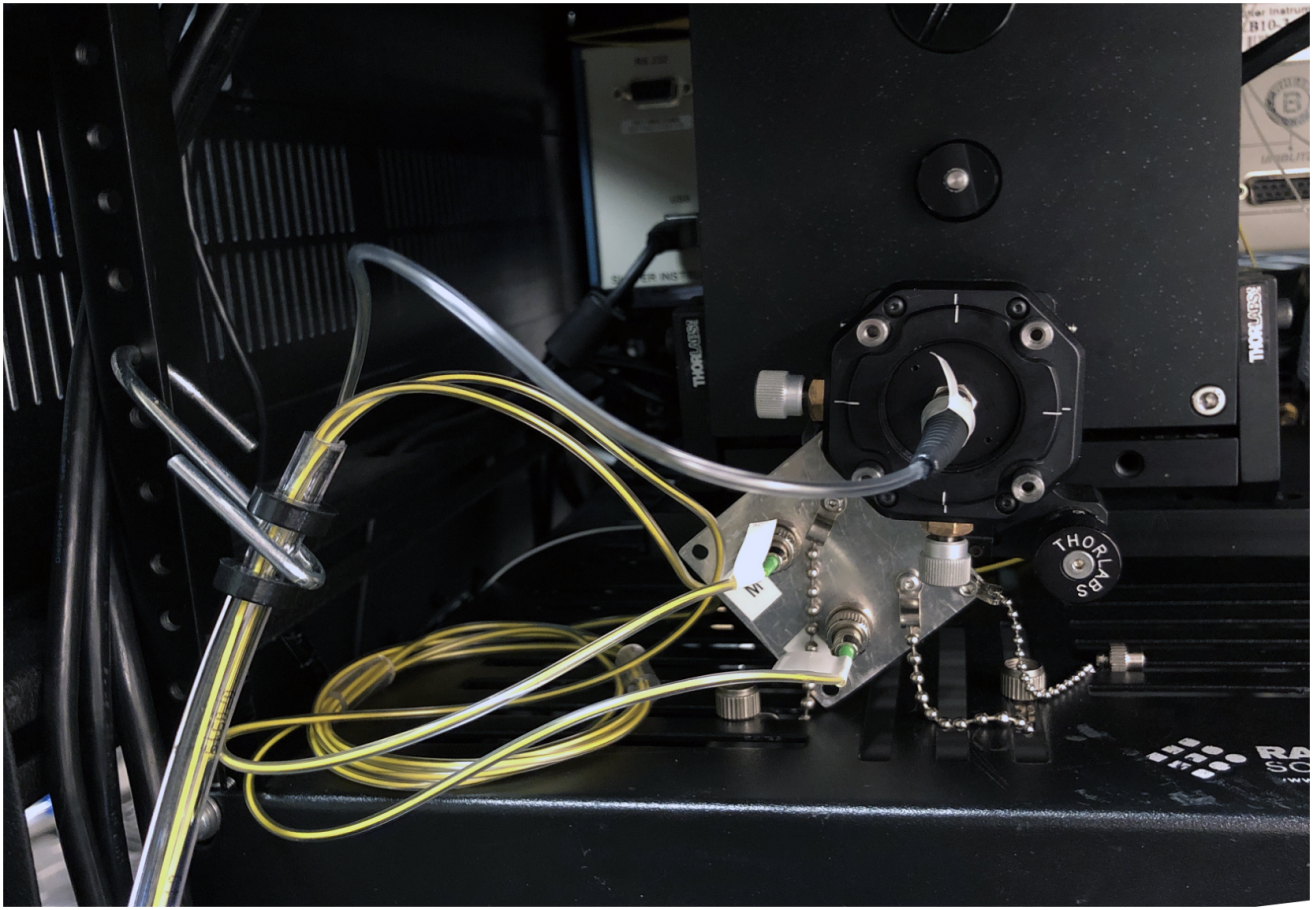
Close up of the protected proximal fibers bridging the medical rack and the endoscope’s vinyl tubing.

**Fig. 6:**
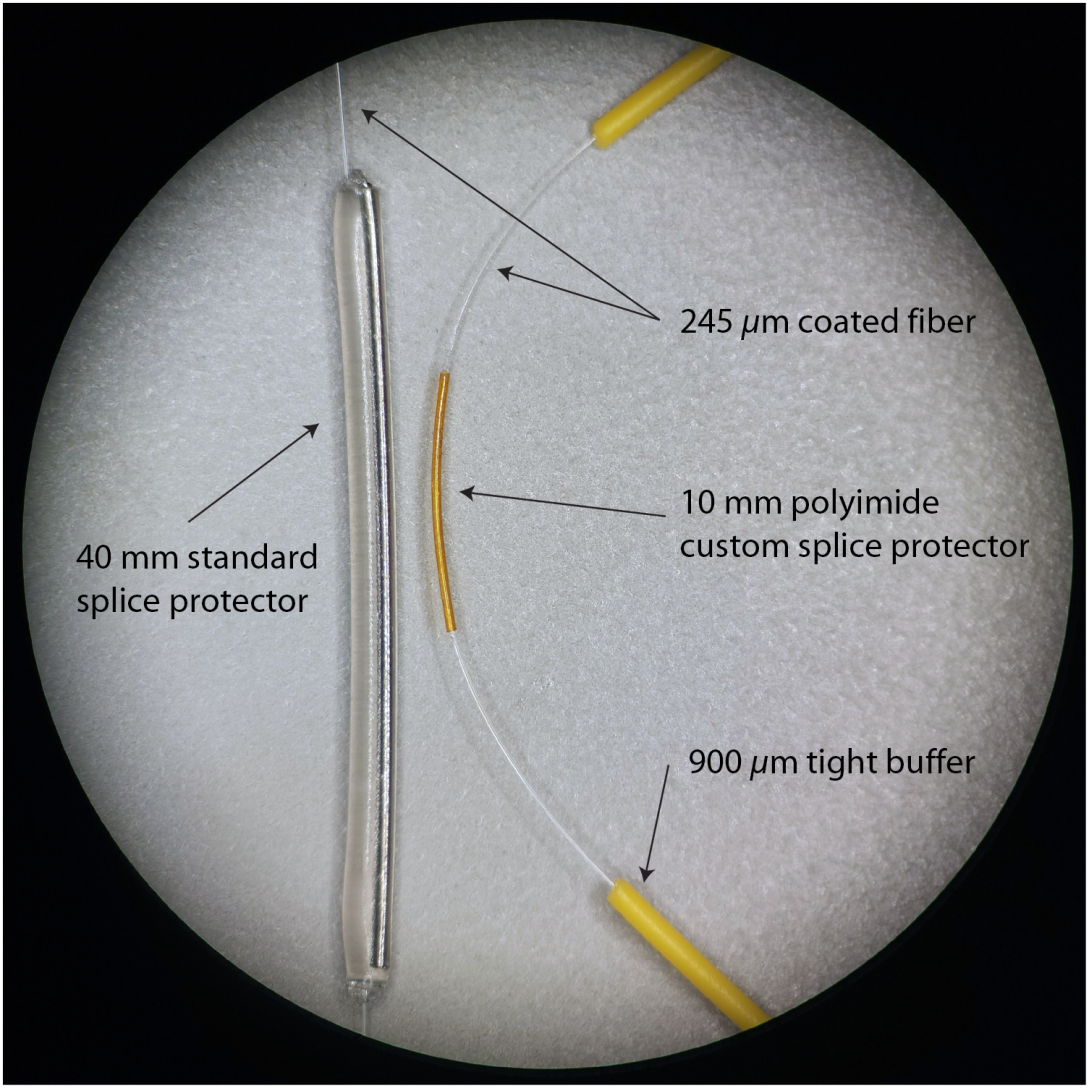
Standard splice protector has a long rigid rod creating a rigid to flexible transition that may become a breaking point at the splice junction. The custom splice protector is semi-rigid and shorter and has eliminated breakage at the splice junction.

The proximal length of the microendoscope consists of a vinyl tube that contains the illumination fiber, imaging fiber bundle, and OCT fiber. It is permanently glued to the handle and is secured to the medical instrument cart during use. Unfortunately, the vinyl has a high friction coefficient and can cause a fiber splice to break if the fibers are accidentally pulled by the tubing itself when bent or unbent. Also, during sterilization and pre-operation setup, the end of the tubing is unattached, so if the fibers are pulled, the tensile forces propagate through the glass fiber core/cladding, a mechanical weakness of glass. Our mitigation strategy, as shown in Fig. 5, was adding additional smaller tubes of vinyl around the fibers where they proximally exit the vinyl tubing. These tubes act as a tight buffer that limits bend radii, eases stress on the splice points, and makes for convenient handling and storage of the fibers. Another increase in robustness was with the addition of splice junction reinforcements, as shown in Fig. 6, which consisted of a snug fitting polyimide tubing (Microlumen, 110-I) and adhesive (Loctite^®^ 4013 cyanoacrylate liquid adhesive) and prepping the surface by cleaning the substrate and adherent with acetone and then prepping the surfaces with primer meant for adhering low-energy plastics (Loctite^®^ 7701™ Prism^®^ medical device adhesive primer), which increased the tensile strength of the bond by ∼150% compared with a fusion splice alone.

**Fig. 7:**
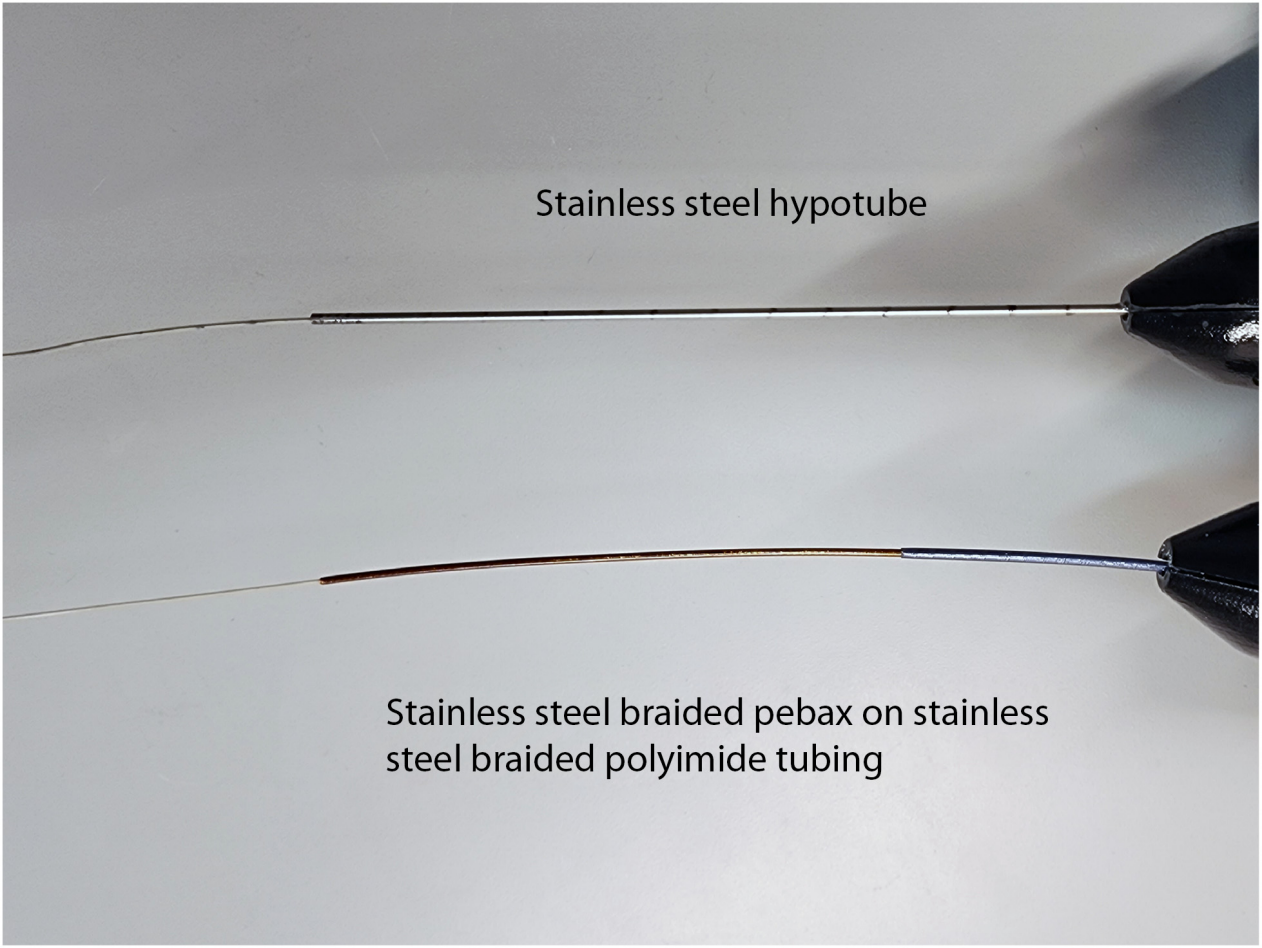
Top: the falloposcope’s proximal working length was initially designed with a SS hypotube to provide a surface for catheter components to slide onto. This created a rigid to flexible transition that was a kink point. Bottom: the new version has a two-layered approach to go from a more rigid braided PEBA elastomer to a semi-rigid braided polyimide section serving the same function while also acting as a crush-resistant strain relief for the fiber.

In previous distal tip/handle designs, the PEEK MLE was glued inside a 304 stainless steel (SS) hypotube of 0.0730″ diameter and 14 cm length extending from the handle. The purpose of the hypotube was to strengthen the transition from the handle to the thin flexible insertable portion of the falloposcope and to provide a larger diameter and crush resistant section for locking the Tuohy Borst valve. However, the rigid hypotube in turn created a failure point at the transition from its rigid end to the flexible falloposcope. Our modification, as shown in Fig. 7, was to replace the hypotube with two layers of medical grade tubing. The larger tube of shorter length was a custom construction of polyether block amide (PEBA) elastomer, an SS braid, and a polytetrafluoroethylene (PTFE) liner. The smaller tube of longer length was constructed of polyimide, an SS braid, and a PTFE liner (Microlumen, 390-VII). The new design is crush resistant yet flexible enough to provide strain relief to the working length of the microendoscope. Another rigid to flexible junction is at the very distal tip where the MLE meets the SS ferrule that is used for bonding the fibers and pull wires in place. Operational adjustments, including the new clear-tipped introducing catheter, which helps avoid forcing the falloposcope tip against uterine tissue, have eliminated failures at this location. However, a more robust solution may be to eliminate the ferrule by bonding the fibers, distal tip optics, and pull wires directly to the PEEK MLE material. Glass-PEEK bonding can be accomplished with Loctite^®^ 4013 cyanoacrylate liquid adhesive;^23^ however, SS pull wires would likely require a non-adhesive method of distal fixation.

### 3.4 Optimization

The falloposcope software originally consisted of a combination of laboratory-written code for OCT (Visual Studio C++, Microsoft, Redmond, Washington, United States) and vendor software for the MFI imaging (Ocular, QImaging, Surrey, BC Canada), which performed well in the laboratory. A detailed standard operating procedure was written for the falloposcope instrument cart operator for obtaining images and communicating with the surgeon. The time restrictions and stress of the OR environment brought out the limitations of both the laboratory-written software and the vendor software under unexpected conditions. To fix bugs, errors and vulnerabilities of our laboratory-written software, we encouraged research personnel to provide code reviews and feedback. We improved the program’s quality, reliability, and functionality by

• providing a warning instead of crash if the laser source is not powered;
• disabling the scan button until the current scan has been completely saved, preventing crash;
• adding a save status bar to give the user peace of mind that the file was saved;
• restricting the GUI windows to a fixed size, preventing possible crash; and
• adding the ability to change the falloposcope-specific calibration file within the program, speeding the switch from using the primary unit to the secondary unit if necessary.

All of these changes to the falloposcope software both expedited and standardized the image-collection process, which is crucial in the OR.

### 3.5 Fiber Bundle

**Fig. 8:**
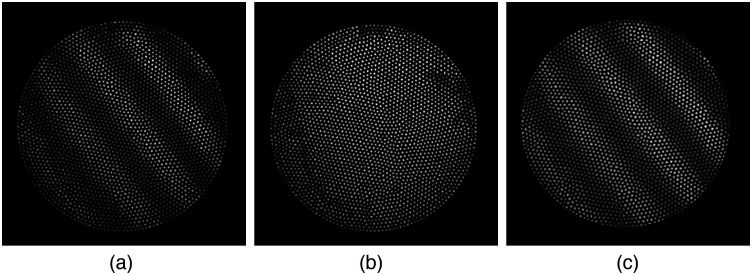
(a) The raw image is of an opal diffusing glass backed Ronchi ruling. (b) The flat field image of the fiber bundle shows that the relative illumination from core to core is not uniform. Using a flat field corrected test image (c), panels (a)–(c) are normalized for easier viewing.

The fiber bundle is made of 3000 fibers that are melted together and are coherent in that each core on one end carries its signal to the other end with minimal crosstalk and in the same relative spatial position. It is used as a relay system to carry the image created by the distal GRIN lens to a medical instrument cart, where a microscope objective and camera lens magnify the image onto the camera sensor. Unfortunately, the individual fibers are not uniform in loss, and variances in intensity occur across the entire bundle, as well as between each falloposcope. To mitigate this variation, flat field images were taken by securing an opal diffusing glass at the falloposcope’s working distance (WD) 5 mm that was back illuminated by a 150 watt quartz halogen source (Dolan-Jenner, Fiber-Lite Mi-150) with single gooseneck fiber optic cable (Dolan-Jenner, BGG1818) to create a near Lambertian source. Test images were obtained of a Ronchi ruling on white opal diffusing glass in the same configuration. Dark images were taken under zero illumination conditions. The test images, R, are corrected by a flat field correction by

$$C=\frac{(R-D)\times {\text{mean}}_{\left(F+p\right)}}{\left(F+p\right)}\times M_{\text{circle}}$$

where C is the flat field corrected image. D is the master dark, which consists of the pixel averaged frame of at least ten dark images. F is the master flat, which is the pixel averaged frame of at least 10 flat field images with the master dark subtracted. A pedestal, p, is added to avoid having extremely low values in the denominator. The average value of the master flat with added pedestal in denoted as ${\text{mean}}_{\left(F+p\right)}$. Finally, $M_{\text{circle}}$ is a circular mask that is applied to the region of interest, which is the portion of the camera image that contains the fiber bundle. The flat field correction is shown in Fig. 8.

### 3.6 GRIN Lens and Stray Light

**Fig. 9:**
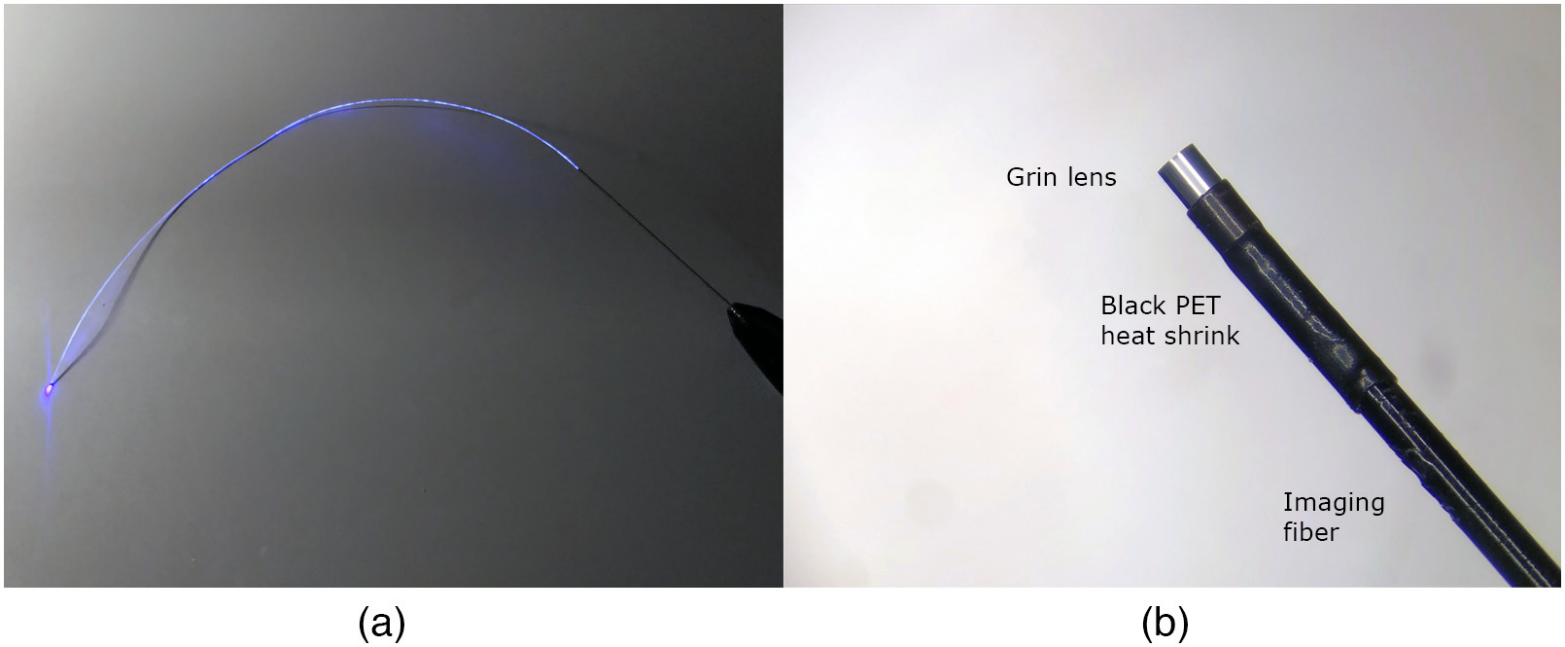
(a) Light leaks out of the thin cladding bare fiber and into fluorescent PEEK MLE. (b) Our solution was to replace the clear heat shrink covering the OD of the GRIN lens with black heat shrink.

Other improvements were made to reduce the background noise, thus improving the signal-to-noise ratio of weak autofluorescence. The first source of noise was caused by stray illumination light coupling into the imaging GRIN lens. The initial prototype illumination fiber (Polymicro FSU 80/85/100 core/cladding/coating) was selected for its small OD, high 0.66 numerical aperture (NA), and high hydroxyl composition enabling efficient propagation of visible laser illumination through the working length of the microendoscope. This fiber was spliced to a 50/125/250/900 core/cladding/coating/buffer OM3 GRIN MMF, which was connected to the output fiber from the beam combiner in the instrument cart. Unfortunately, the polymicro fiber became obsolete before the beginning of the pilot study. Its selected replacement was a custom bare fiber (Fiber Technology Incorporated, 85/100 core/cladding bare borosilicate fiber), which retained the small diameter necessary to traverse the MLE. However, because the custom bare fiber did not have a coating, it was more likely to suffer from fine cracks along the surface due to micro bending, compression, and tension as the fiber was handled during installation. The telltale sign of these fine cracks was illumination light leakage out along almost the entire working length, as shown in Fig. 9. The leaked light transmits directly into the surrounding PEEK, causing autofluorescence of the material. Some of the PEEK autofluorescence was collected by the GRIN lens and propagated through the fiber bundle, causing a high background signal in the images. Our solution to this stray light problem was to use blackened PET heat shrink to hold the GRIN lens to the imaging fiber bundle, blocking stray light collection. Future iterations of the falloposcope will utilize black-colored polymer, assuring that any stray light and background autofluorescence is absorbed.

### 3.7 Proximal Imaging System Optimization

Additional background noise was discovered in the medical instrument cart’s proximal imaging system. This system begins at the SMA905 port where the microendoscope’s imaging bundle is connected. The bundle’s cores are imaged onto a sensor after passing through a filter wheel. The open cage system allowed bright illumination in the OR to couple into the imaging system. The unwanted background was resolved by closing the cage system with black panels and threaded barrels when possible.

**Fig. 10:**
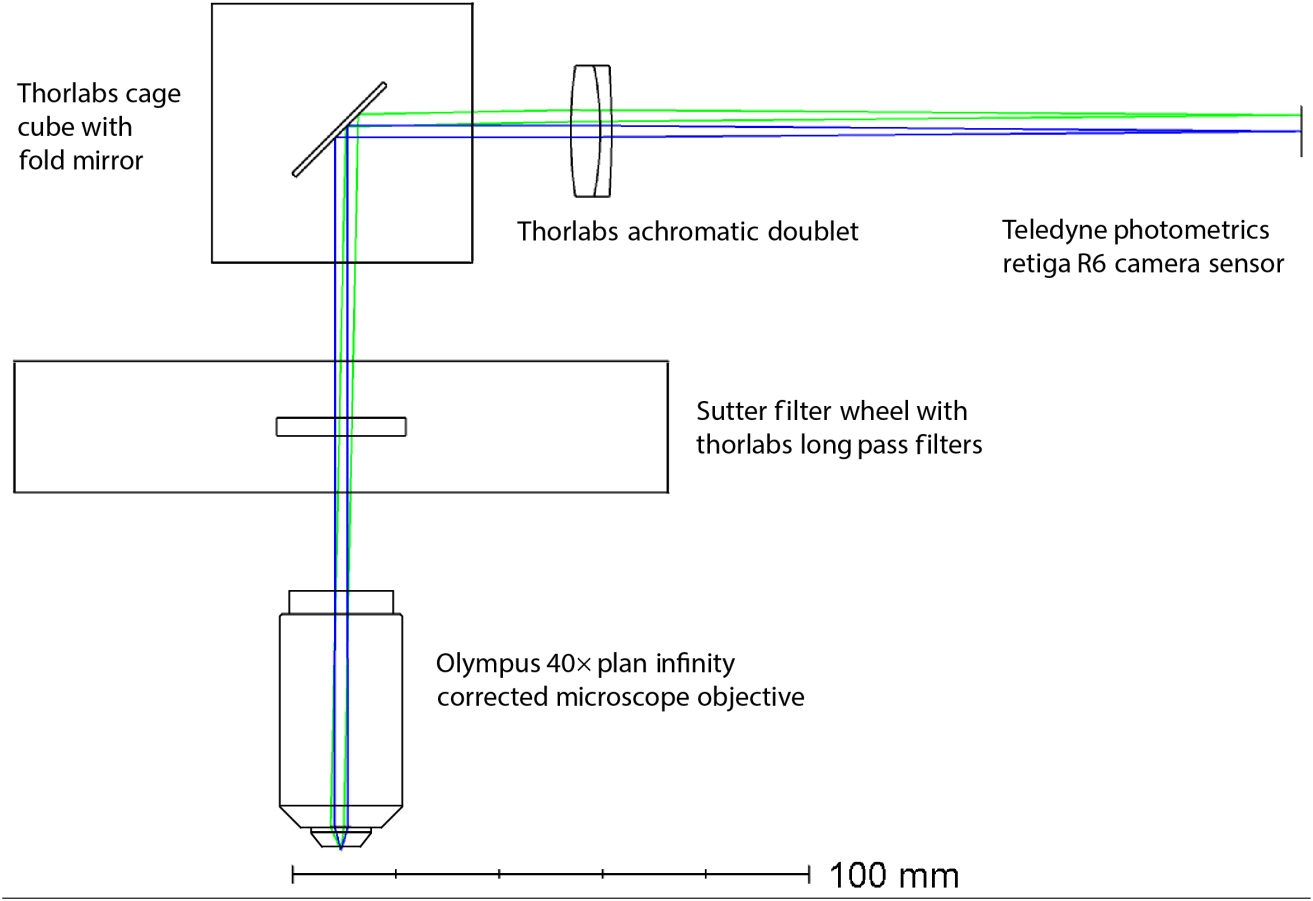
Simplified representation of the proximal imaging system in the medical cart to show how light from the imaging fiber bundle is collimated, filtered, and then focused onto an image sensor.

Further optimization was made to the proximal imaging system. The benchtop prototype system utilized a ultra-violet (UV) achromat (OptoSigma, NUDL-30-150P) as the camera focusing lens because this system included a 275 nm laser source. It was necessary to change the position of the objective when images were taken with various light sources between 275 and 638 nm, as well as when a filter was introduced to the optical path for fluorescence images. In the updated clinically ready system, the sources selected were 405, 488, 520, and 642 nm, with UV avoided due to safety concerns of applying this light to reproductive tissue. The UV achromat remained in the system because the aberrations caused by it were not significant except for chromatic focal shift, which could be compensated by changing the objective position. The limited 15 min clinical time, however, made changing the objective position for every measurement impractical. The UV achromat was replaced with a better performing visible (VIS) achromat (Thorlabs, AC254-150-A), and a broadband (${\mathrm{MgF}}_{2}$) coated blank (Edmund Optics, No. 32-951) was installed into the filter wheel for reflectance measurements. These changes allowed us to collect reflectance and fluorescence measurements without having to change the objective’s position. The proximal imaging system optical design is shown in Fig. 10. Finally, the entire proximal imaging system was secured onto a small platform with shock absorbing Sorbothane feet (Thorlabs AV3) to ruggedize for transport to the OR.

### 3.8 Illumination System

The goal of our illumination system is to have a large angle, top-hat beam profile from the perspective of the imaging system, so the object plane has nominal illumination uniformity. The small size of the endoscope permits only one illumination fiber, leading to asymmetric illumination and making a large angle illumination more critical at the short WDs employed. The illumination fiber’s 0.66 NA provides an ∼83  deg illumination angle, sufficient to cover the field of view of the image system in the minimum WD of 5 mm in air or 6.7 mm in 0.9% saline solution. The illumination laser sources are coupled into SMFs with small NAs. Once the SMF is coupled to the falloposcope’s illumination fiber, mode mixing occurs and the output beam profile broadens. However, the length of illumination fiber, 1 m, is not long enough to provide complete mode mixing and a flat-top beam output. The first falloposcope prototype used a miniature mode scrambler to force microbends in the illumination fiber to promote rapid mode-mixing. This system was effective and had the added benefit of permitting a varying amount of mode scrambling and consequently output beam NA. However, the microbends significantly reduced the longevity of the fiber, resulting in failures. The second fiber selected was connected to the OM3 by the manual splice method, which in turn meant that mode-scrambling was no longer necessary as the accidental axial position mismatch that occurred during manual splicing led to rapid, albeit lossy, mode-mixing. When the new third illumination fiber was selected, we wanted to avoid utilizing the mode scrambler, so it was decided to exchange the OM3 GRIN MMF for an SMF-28 fiber for better fiber-coupling. The beam profile of the SMF-28 is Gaussian, and splicing it to the 0.66 NA borosilicate fiber creates a broad and smooth, albeit non-uniform, Gaussian profile on the tissue surface. To assure comparable performance of all falloposcopes, the current of each laser sources is adjusted to normalize the output power, controlling for varying losses from endoscope to endoscope.

### 3.9 Initial Performance with Improved Prototype

**Fig. 11:**
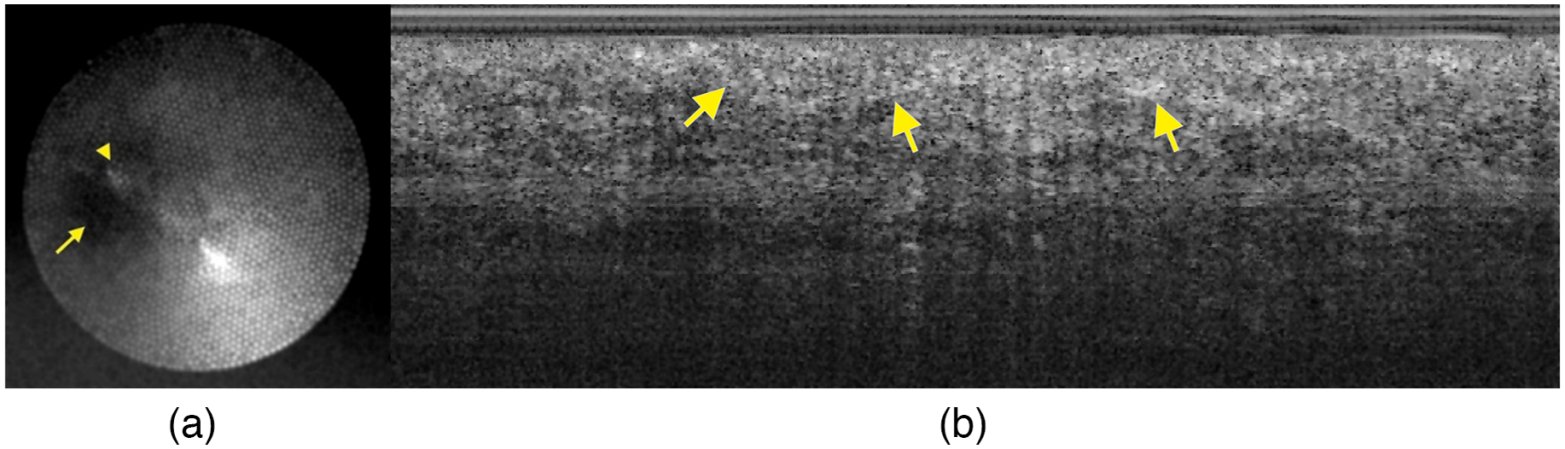
(a) Example reflectance image obtained with 488 nm laser illumination. The lumen (arrow) as well as a protrusion in the wall (arrowhead) are shown. The high reflectance streak in the center is specular reflectance. Gaussian blur is applied to reduce the honeycomb artifact. (b) Example OCT image, with overlapping FT plicae (arrows show examples). Histogram adjusted to increase contrast.

The improvements made to the falloposcope system eliminated points of failure and enhanced operational usability. The modifications to the system successfully allowed the full 15 min of allowable OR time to be utilized, thus increasing the quantity of data acquired. To be classified as non-significant risk, this study was performed on volunteers with no evidence of, or elevated risk for, ovarian cancer. Therefore, we cannot yet evaluate the falloposcope’s diagnostic potential. However, preliminary *in vivo* imaging capabilities are shown in Fig. 11. A reflectance image with 488 nm illumination, extracted from an in vivo video recording, is shown in Fig. 11(a) revealing the FT lumen as well as a protrusion in the wall of the FT. An OCT image of the proximal FT is shown on the right, revealing overlapping FT plicae.

*Ex vivo* imaging has been performed with the falloposcope and some initial images published.^21^ The resolution of the forward looking imaging system is severely limited by the 3000 pixels of the miniature imaging fiber bundle; thus these images are a much lower visual quality than those taken previously with a tabletop MFI system.^14^ However, *ex vivo* and *in vivo* multispectral fluorescence and reflectance images taken with the falloposcope are comparable.

Falloposcope OCT images are similar in imaging depth and axial resolution to *ex vivo* images obtained with commercially available OCT tabletop systems. However, falloposcope images are lower lateral resolution due to the small diameter of the falloposcope and a conservative choice of NA to assure a long depth of focus. Most importantly, the falloposcope has no distal scanning mechanism, and 2D images are obtained by manual pull back. This method can cause repeated or discontinuous a-scans due to non-uniform pull back speed, a situation that is exacerbated in *in vivo* versus *ex vivo* imaging. In the future, we intend to implement an automated pull back system to provide a constant speed through acquisition.

We have described the iterative prototyping process utilized to transition the engineering design work from the laboratory to the clinical environment. The changes made to the microendoscope system, driven by stakeholder input, resulted in significant operational success. Notably, we have not experienced any broken fibers in the falloposcope during sterilization, pre-procedure operations, or normal *in vivo* use after implementing the design changes stated in this manuscript. We have plans for future incremental upgrades to the overall system, including designing a smaller profile and lighter weight handle, improving the fiber protection system, and developing a variant of the falloposcope that collects cells for cytology.

## Code, Data, and Materials Availability

Standard operating procedures for endoscope assembly are available from the authors upon request.
